# Genistein Enhances GLUT4 Expression and Translocation in the Gastrocnemius Muscle and Improves Systemic Glucose Metabolism in Ovariectomized Mice

**DOI:** 10.3390/nu17172811

**Published:** 2025-08-29

**Authors:** Xiaomeng Yang, Kun Dai, Suqing Wang

**Affiliations:** 1 School of Public Health, Wuhan University, Wuhan 430223, China; 2022203050022@whu.edu.cn; 2 School of Nursing, Wuhan University, Wuhan 430223, China; 2024103070003@whu.edu.cn

**Keywords:** estrogen, diabetes, bioactive compounds, skeletal muscle, liver

## Abstract

**Background**: Premenopausal women typically exhibit superior glucose metabolism compared to males, but this metabolic advantage is lost after menopause. The primary cause is the sharp decline in estrogen levels post-menopause. Genistein, a natural compound predominantly derived from leguminous plants, possesses structural similarity to estrogen. This enables specific binding to estrogen receptors, allowing genistein to exert estrogen-mimicking effects under conditions of estrogen deficiency. The aim of this study was to investigate the effects and potential mechanisms of genistein on glucose metabolism in the liver and skeletal muscle of ovariectomized (OVX) mice fed a high-fat diet (HFD). **Methods**: Animal experiments were performed using 8-week-old mice that were OVX to construct a model of estrogen deficiency and impaired their glucose metabolism by a continuous HFD. Genistein was administered by gavage (50 mg/kg-day) for 10 weeks and 17β-estradiol was administered subcutaneously (50 μg/kg) every 4 days for 10 weeks as a positive control. **Results**: Genistein significantly improved glucose metabolism (including fasting glucose, postprandial glucose, serum glucose levels, and HOMA-IR index) but did not affect serum estrogen levels and uterine weights in OVX mice. Genistein promoted increased expression and translocation of glucose transporter 4 (GLUT4) in the gastrocnemius muscle, enhanced phosphorylation of the PI3K/AKT pathway, and upregulated expression of the G protein-coupled estrogen receptor (GPER). Concurrently, it stimulates hepatic glycogen accumulation and upregulates GLUT2 expression in the liver. **Conclusions**: GEN improves glucose metabolism in ovariectomized mice, and this improvement is primarily attributed to increased expression and membrane translocation of GLUT4 in the gastrocnemius muscle mediated by the GPER-PI3K/AKT pathway.

## 1. Introduction

Studies have shown that premenopausal women generally have higher insulin sensitivity and lower incidence of type 2 diabetes (T2D) compared to men of the same age [1]. However, this metabolic advantage gradually disappears after menopause, and one of the key factors is a significant reduction in circulating levels of 17β-estradiol (E2) [2]. Data from clinical studies have shown that postmenopausal women receiving hormone replacement therapy (HRT) show a significant trend toward a decrease in the degree of insulin resistance and cumulative incidence of diabetes mellitus [3]. Of 14 studies evaluating the effects of estrogen therapy on postmenopausal women’s beta-cell function, more than 50% reported significant improvement in glucose-stimulated insulin secretory function [4].

Although HRT significantly improves metabolic abnormalities (e.g., insulin resistance, diabetes risk) in postmenopausal women, its long-term safety warrants caution. The Women’s Health Initiative (WHI) study found that oophorectomized women receiving conjugated equine estrogens (CEEs) had a lower breast cancer risk versus placebo (HR = 0.78). Conversely, women receiving CEE–medroxyprogesterone acetate showed increased breast cancer risk (HR = 1.28) [5]. HRT provides cardiovascular benefits during early atherosclerosis but may increase plaque vulnerability in advanced vascular aging, triggering thrombosis [6]. Oral HRT elevates venous thromboembolism (VTE) risk, whereas transdermal HRT poses no significant VTE risk [7]. Based on these potential risks, we would like to look for safer alternative substances with a view to minimizing adverse effects while improving abnormalities in glucose metabolism in postmenopausal women.

Genistein (GEN), the most potent active factor of soy isoflavones, is an important candidate for replacement because of its chemical structure, which is similar to that of estrogen [8]. In estrogen deficiency, GEN can mimic or modulate endogenous estrogen by binding to estrogen receptors and is used to treat a variety of disorders associated with impaired estrogen secretion [9]. When estradiol levels are high, GEN can act as an estrogen receptor antagonist to reduce the risk of certain solid tumors [10]. Numerous recent studies have demonstrated that GEN, either alone or as a dietary supplement, is useful in the treatment or prevention of diabetes and related complications [11,12,13,14]. For example, it can reduce inflammation-induced diabetes by decreasing TNFβ secretion through activation of ERK- and p38 MAPK-dependent pathways and protect against dexamethasone-induced apoptosis of pancreatic β-cells by decreasing TRAIL and DR5 protein expression [15,16]. Of note, a follow-up study in Korea demonstrated gender specificity between GEN intake and T2D risk; women’s GEN intake negatively correlated with a significant dose–response effect on T2D, whereas men’s did not [17]. To date, however, the effects of GEN on glucose metabolism in the absence of estrogen and the mechanism of action of GEN are unknown.

The liver and skeletal muscle are the predominant organs of energy metabolism in rodents, taking up excess glucose from the blood and converting it to glycogen for storage. Since the plasma membrane is impermeable to polar molecules such as glucose, cellular uptake of glucose is accomplished through membrane-associated carrier proteins. Glucose transporter protein 2 (GLUT2) and glucose transporter protein 4 (GLUT4) are the major transporter proteins in liver and skeletal muscle, respectively [18]. Of these, GLUT4 is mainly dependent on the insulin cascade reaction for activation, which in turn translocates to the plasma membrane to function. Recently, it was found that estradiol significantly increased the expression of GLUT4 and insulin receptor substrate (IRS1), but not phosphorylated insulin receptor substrate (P-IRS1), in cardiac muscle of ovariectomized mice [19]. This suggests that estrogen may be able to regulate the expression of GLUT4 through other pathways by increasing the activation of the downstream pathway of the insulin cascade response. For example, both membrane-embedded estrogen receptor and estrogen-associated G protein-coupled receptor (GPER) can activate the PI3K/Akt pathway upstream of GLUT4 [20]. Therefore, the aim of the present study was to investigate the effect of oophorectomy on the abnormalities in glucose metabolism induced by high-fat feeding and the possible mechanism by which GEN improves the metabolism of glucose in the absence of estrogen.

## 2. Materials and Methods

### 2.1. Animal Preparation

All procedures for animal handling complied with the ethical requirements for laboratory animal welfare at the Animal Testing Center/ABSL-III Laboratory of Wuhan University and followed the National Guidelines for the Care and Use of Laboratory Animals. Female C57BL/6 mice (7 weeks old) were housed in a temperature- and humidity-controlled barrier environment with a 12 h light/dark cycle and free access to food and water. Ovariectomy was performed one week after acclimatization feeding. Mice were anesthetized with sodium pentobarbital (35 mg/kg) and placed on a dissection pad; the skin was exposed by alcohol disinfection, and a small incision was made laterally with ophthalmic scissors at the left margin of the dorsal spine. The tissue was gently peeled back, the fat pads surrounding the ovaries were removed, the ovarian artery was ligated, and the ovaries were excised. The remaining tissues were retracted, and the incision was closed with sutures. Sham-operated mice were anesthetized to open the incision without any treatment; they were then sutured to close the incision and rearing was resumed.

### 2.2. Experimental Groups

**Figure 1:**
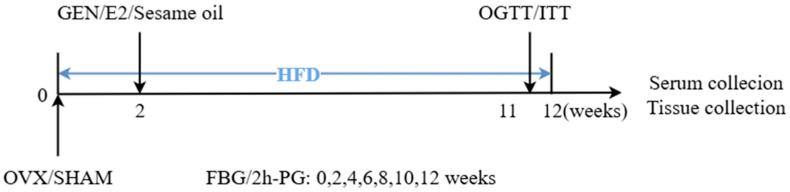
Experimental design and animal grouping. OVX or SHAM surgery was performed, followed by feeding on HFD. Intervention began at 2 weeks post-surgery. At 12 weeks post-surgery, mice were euthanized, and serum and tissues (liver, gastrocnemius muscle, tibialis anterior muscle, abdominal fat, and uterus) were collected. Fasting blood glucose and 2 h postprandial glucose were measured every two weeks. Mouse body weight was measured weekly. HFD was replaced daily, and the weight of the HFD was recorded.

At 2 weeks post-ovariectomy (OVX) or sham surgery, mice were randomized into four groups: (1) SHAM (*n* = 9): sham-operated mice were fed a high-fat diet (HFD; 45% fat, Shulaibao Biotech, Wuhan, China) and received daily gavage with 0.2 mL corn oil. (2) OVX (*n* = 9): bilaterally ovariectomized mice were fed an HFD and received daily gavage with 0.2 mL corn. (3) GEN (*n* = 10): OVX mice were fed an HFD and received daily gavage of GEN (50 mg/kg/day, Tauto Biotech, Shanghai, China) [21,22,23]. (4) E2 (*n* = 10): OVX mice were fed an HFD and administered subcutaneous injections of E2 (50 μg/kg, twice weekly; Yeasen Biotechnology, Shanghai, China) [24,25]. Table 1 presents the information on the groups simply and clearly. Body weight and food intake were monitored weekly throughout the study. Schematic representations and chronological timelines of groupings, experimental treatments, and mouse euthanasia procedures are depicted in Figure 1.

### 2.3. Glucose Metabolism Indicators

#### 2.3.1. Measurement of Fasting and 2 h Postprandial Blood Glucose

At 9:00 p.m., feed was removed from the mice, followed by replacement of bedding in the cage. After 12 h of removal of food, but not of drink, the glucose concentration in the tail-tip blood of the mice was measured using a glucose meter (Accu-Chek Guide, Germany) and recorded as fasting blood glucose (FBG). At the end of the FBG measurements, the mice were gavaged with glucose in aqueous solution (2 g/kg) and then released back to the cages, and then the glucose concentration in the tail-tip blood of the mice was measured again 2 h later and recorded as 2 h postprandial glucose (2h-PG).

#### 2.3.2. Assessment of Insulin Resistance

Insulin resistance was evaluated using the homeostasis model assessment (HOMA) [26]. Specifically, the HOMA-IR score was calculated with the following formula: HOMA-IR = (fasting glucose (mmol/L) × fasting insulin (μU/mL))/22.5.

#### 2.3.3. Oral Glucose Tolerance Test and Insulin Tolerance Test

An oral glucose tolerance test (OGTT) and an insulin tolerance test (ITT) were performed 1 week before euthanasia to assess baseline glucose tolerance and insulin sensitivity, as well as at the end of the intervention. Following a 12 h fast, all mice were administered glucose solution (2 g/kg) by oral gavage. Tail blood was collected at 0, 30, 60, 90, and 120 min post-administration for glucose measurement using a glucometer (Accu-Chek Guide, Basel, Switzerland). The area under the curve (AUC) for glucose was calculated. Three days after the OGTT, mice fasted for 4 h received intraperitoneal injections of human insulin (0.75 U/kg). Tail vein blood sampling and glucose measurements were performed at identical time points (0, 30, 60, 90, 120 min) post-injection, followed by AUC quantification.

### 2.4. Glycogen Content Measurement

Glycogen content in the liver and gastrocnemius muscle was quantified using the Glycogen Content Assay Kit (Solarbio, Wuhan, China), following the manufacturer’s instructions.

### 2.5. RNA Extraction and Quantification Using Real-Time Polymerase Chain Reaction (qPCR)

Total RNA was extracted from the liver and gastrocnemius muscle using an RNA extraction solution (Servicebio, Wuhan, China) according to the manufacturer’s protocol. Reverse transcription was performed using Hifair^®^ III 1st Strand cDNA Synthesis SuperMix for qPCR (gDNA digester plus) (Yeasen, Shanghai, China) and a 20 μL reaction volume containing 0.5–2 μg of sample RNA. PCR was performed using Hieff^®^ qPCR SYBR Green Master Mix (No Rox) (Yeasen, Shanghai, China) in a 10 μL volume with a concentration of 0.05 μM for each primer. Primer sequences are shown in Table 2.

### 2.6. Western Blot Analysis

**Figure A1:**
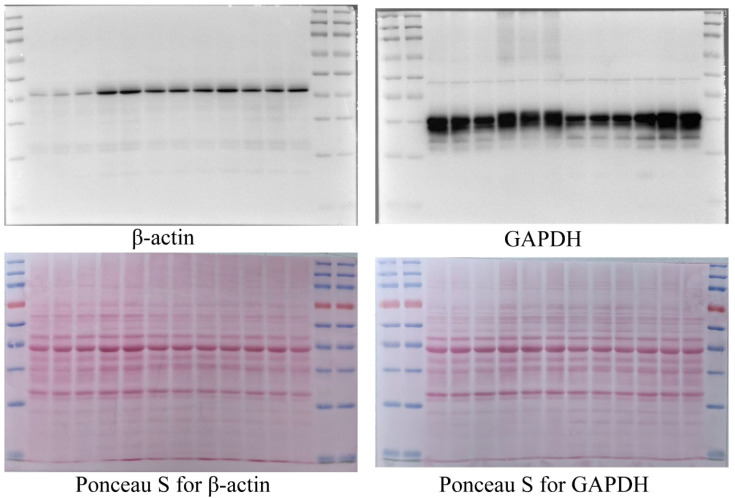
Internal reference controls and total protein normalization.

A 1:100 mixture of protease phosphatase inhibitor and RIPA lysate (Epizyme, Nanjing, China) was used to extract total protein from the liver and gastrocnemius muscle according to the manufacturer’s instructions. Protein samples were separated on 10% and 8% sodium dodecyl sulfate–polyacrylamide gel electrophoresis gels and transferred to polyvinylidene difluoride membranes. The membranes were blocked with 5% skimmed milk for 1 h at room temperature, followed by overnight incubation at 4 °C with primary antibodies: anti-GLUT4 (1:2000, Proteintech, Wuhan China), anti-GPER (1:2000, Genetex, Irvine, CA, USA), anti-phosphoinositide 3-kinase (PI3K) (1:1000, Yeasen, Shanghai, China), anti-phospho-phosphoinositide 3-kinase (p-PI3K) (1:1000, Yeasen, China), anti-Akt (1:1000, Yeasen, China), anti-anti-phospho-Akt (p-Akt) (1:1000, Yeasen, China), anti-β-actin (1:5000, Yeasen, China), and anti- glyceraldehyde-3-phosphate dehydrogenase (GAPDH; 1:10,000, Proteintech, China). After washing, the membranes were probed with the corresponding secondary antibody (horseradish-peroxidase coupled IgG). The density of individual protein bands was quantified by optical density measurements of the blots using ImageJ software 1.8.0. The protein expression of GAPDH and actin in muscle samples varied across groups due to the modeling approach affecting glucose metabolism (Figure A1). Thus, we employed Ponceau S staining to quantify total proteins, which serves as a loading control for normalizing protein expression independent of specific markers [27].

### 2.7. Immunofluorescence Staining

Fresh tissues were fixed in 4% paraformaldehyde at 4 °C for 24 h immediately after collection, rinsed under running water for 30 min, and subjected to graded ethanol dehydration, xylene clearing, and paraffin infiltration. The tissues were then embedded in paraffin blocks, solidified at 4 °C, trimmed, and sectioned at 4–5 μm after precooling at −20 °C. The sections were subjected to antigen retrieval in EDTA buffer (pH 8.0) using microwave irradiation for 25 min, washed, and then blocked with serum. After washing, the following secondary antibodies (FITC-conjugated goat anti-mouse IgG and CY3-conjugated goat anti-rabbit IgG; Proteintech, China) were added and incubated for 50 min at room temperature in the dark. Subsequently, the primary antibody against β-catenin was added, and the above steps were repeated. After washing, the nuclei were counterstained with DAPI. The slices were sealed with an anti-fluorescence quenching sealer, observed under a fluorescence microscope and images were captured. Immunofluorescence intensity and co-localization ratio were counted using ImageJ software.

### 2.8. Statistical Analysis

Statistical analyses and data visualization was conducted using GraphPad Prism 8.0. One-way Analysis of Variance (ANOVA) or repeated-measures ANOVA was used for group comparisons. If ANOVA was significant, Dunnett’s test was used to compare the treatment groups (SHAM, GEN, E2) vs. OVX. Data are expressed as mean ± standard deviation. Significance was set at *p* < 0.05.

## 3. Results

### 3.1. Unlike E2, GEN Does Not Alter Estrogen Levels or Uterine Atrophy

**Figure 2:**
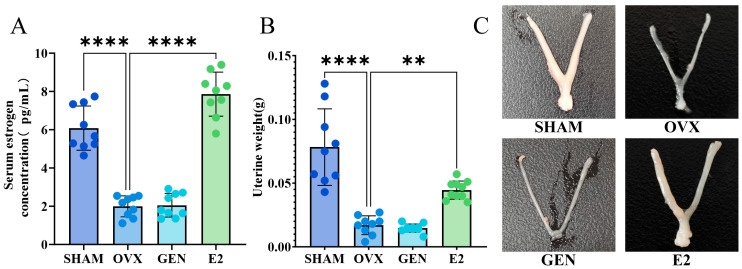
Estrogen levels and uterine size in mice at 12 weeks after OVX. (**A**) Serum estradiol; (**B**) uterine weight; (**C**) uterine morphology. ** *p* < 0.01, **** *p* < 0.0001. SHAM/OVX: *n* = 9; GEN/E2: *n* = 10.

At 12 weeks postoperatively, OVX mice exhibited a significant decrease in serum estrogen levels, accompanied by uterine atrophy, indicating successful replication of the postmenopausal estrogen-deficient state. E2 significantly restored serum estrogen levels and maintained uterine morphology in OVX mice. In contrast, GEN neither altered serum estrogen levels nor improved uterine atrophy in OVX mice, suggesting that GEN does not directly supplement estrogen or act directly on uterine tissue (Figure 2).

### 3.2. GEN Treatment Lowers Abdominal Fat Accumulation in OVX Mice

**Figure 3:**
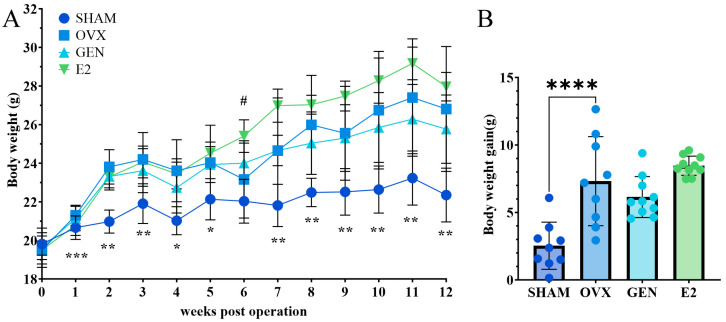
Body weights in mice. (**A**) Body weight trajectory (0–12 weeks post-ovariectomy), * *p* < 0.05 GEN vs. OVX, ** *p* < 0.01 GEN vs. OVX, *** *p* < 0.001 GEN vs. OVX, ^#^ *p* < 0.05 E2 vs. OVX; (**B**) cumulative weight gain, **** *p* < 0.0001. SHAM/OVX: *n* = 9; GEN/E2: *n* = 10.

**Figure A2:**
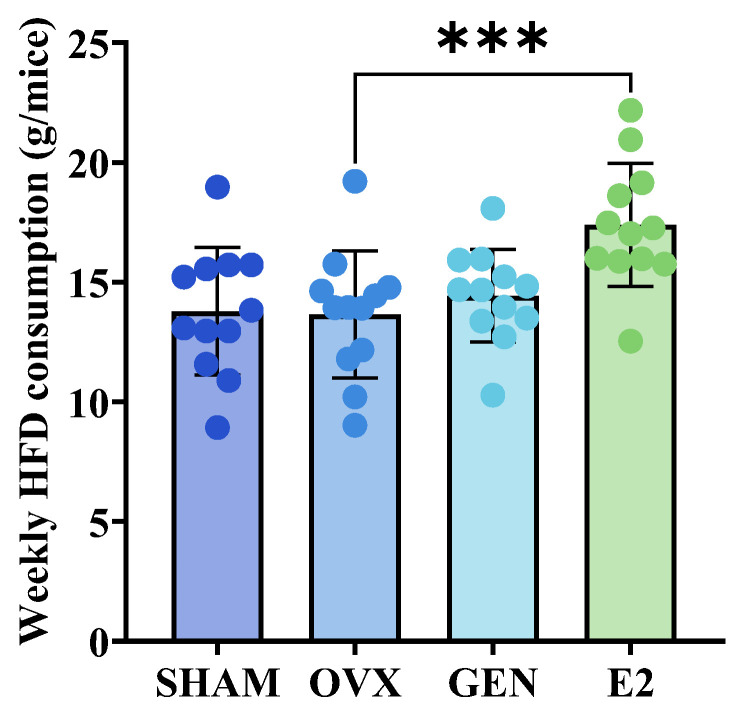
Weekly HFD consumption. *** *p* < 0.001.

OVX mice exhibited greater body weight gain than SHAM mice after 12 weeks of HFD feeding, whereas GEN and E2 administration did not significantly affect body weight (Figure 3A,B). While weight gain differences between E2 and OVX mice did not reach statistical significance, E2 mice demonstrated a consistent weight gain trend. Post-intervention monitoring of weekly HFD consumption indicated elevated intake in the E2 group compared to other cohorts (Figure A2 in the Appendix A). Both GEN and E2 groups showed reduced abdominal fat mass compared to OVX controls. In addition, E2-treated mice developed mildly increased weights of the liver, pancreas, and tibialis anterior muscle compared with OVX mice (Table 3).

### 3.3. GEN Treatment Improves Glucose Metabolism in OVX Mice

**Figure 4:**
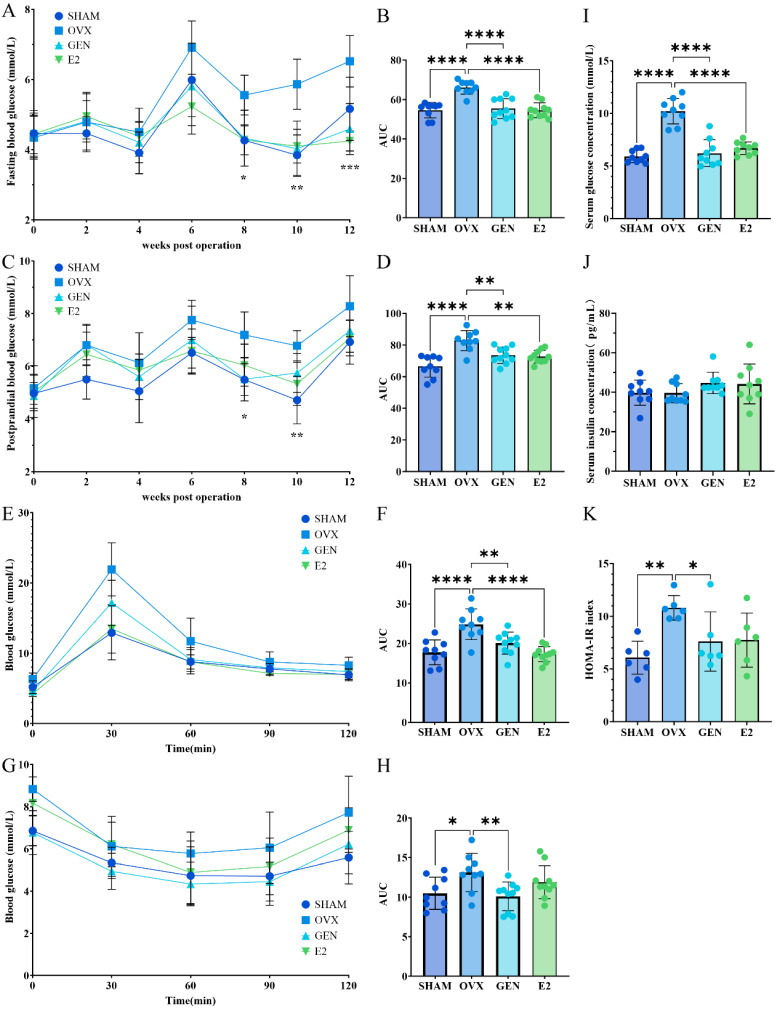
Glucose metabolism in mice. (**A**,**B**) The fasting blood glucose curve and the area under the curve (AUC; 0–12 weeks post-ovariectomy); (**C**,**D**) postprandial blood glucose curve and AUC (0–12 weeks post-ovariectomy); (**E**,**F**) oral glucose tolerance test (OGTT) glucose curve and AUC (0–120 min); (**G**,**H**) insulin tolerance test (ITT) glucose curve and AUC (0–120 min); (**I**) serum glucose; (**J**) serum insulin; (**K**) HOMA-IR index. (**A**,**C**) * *p* < 0.05 GEN vs. OVX, ** *p* < 0.01 GEN vs. OVX, *** *p* < 0.001 GEN vs. OVX; (**B**,**D**,**F**,**H**,**I**,**K**) * *p* < 0.05, ** *p* < 0.01, **** *p* < 0.0001. SHAM/OVX: *n* = 9; GEN/E2: *n* = 10.

After surgery, fasting and postprandial glucose were significantly elevated in OVX mice compared to SHAM mice, and these glycemic impairments were significantly reversed by GEN and E2 treatments (Figure 4A–D). Notably, the fasting glucose of SHAM mice showed a sharp increase at week 12 after OVX, whereas GEN-treated mice maintained relatively stable blood glucose levels. This suggests that the fasting glucose impairment by HFD in female mice may be close to the compensatory threshold, and the estrogen level under normal physiological state can no longer play a sufficient protective role. OVX mice exhibited worse glucose tolerance and insulin tolerance compared with SHAM mice (Figure 4E,F). Both GEN and E2 treatments improved glucose tolerance in OVX mice, but only GEN significantly improved insulin tolerance in OVX mice (Figure 4G,H). Serum glucose levels were higher in OVX mice than in SHAM mice, and GEN and E2 treatments reduced serum glucose levels in OVX mice (Figure 4I). Notably, however, there was no significant difference in serum insulin levels between groups (Figure 4J). Subsequently, we assessed overall insulin sensitivity using the HOMA-IR index and found that insulin sensitivity in OVX mice was only half of that in SHAM mice, but GEN and E2 restored insulin sensitivity in OVX mice to about the same level as in SHAM mice (Figure 4K). These results suggest that long-term treatment of GEN improves glucose metabolism and restores insulin sensitivity in OVX mice.

### 3.4. GEN Upregulates mRNA Expression of GLUT4 and GLUT2

**Figure 5:**
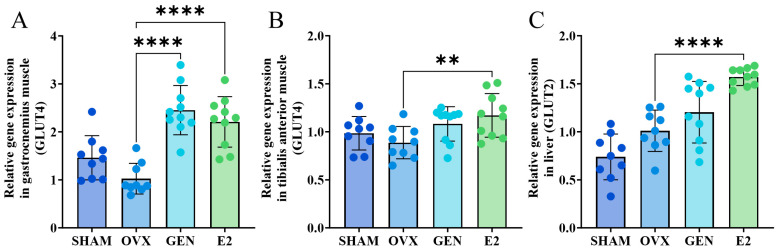
mRNA expression of glucose transporter proteins. (**A**) GLUT4 mRNA expression in gastrocnemius muscle (*n* = 9); (**B**) GLUT4 mRNA expression in tibialis anterior muscle (*n* = 9); (**C**) GLUT2 mRNA expression in liver (*n* = 9). ** *p* < 0.01, **** *p* < 0.0001.

To examine the effect of GEN on glucose intake capacity, we examined mRNA expression of GLUT2 in liver and GLUT4 in skeletal muscle. OVX mice had a 38.3% decrease in GLUT4 in gastrocnemius muscle compared to SHAM mice. GLUT4 expression was increased by 137.1% in the gastrocnemius muscle of GEN mice and 111.2% in E2 mice compared with OVX mice (Figure 5A). GLUT4 expression was increased by 31.5% in the tibialis anterior muscle of E2 mice, but GEN did not affect GLUT4 expression in the tibialis anterior muscle (Figure 5B). Compared with OVX mice, GLUT2 expression was increased by 38.7% in the liver of GEN mice and by 56.9% in E2 mice (Figure 5C). The above results indicate that GEN induced an increase in the expression of glucose transporter proteins in muscle and liver and more significantly increased the expression of GLUT4 in gastrocnemius muscle.

### 3.5. GEN Promotes GLUT4 Expression and Transport in Gastrocnemius Muscle

**Figure 6:**
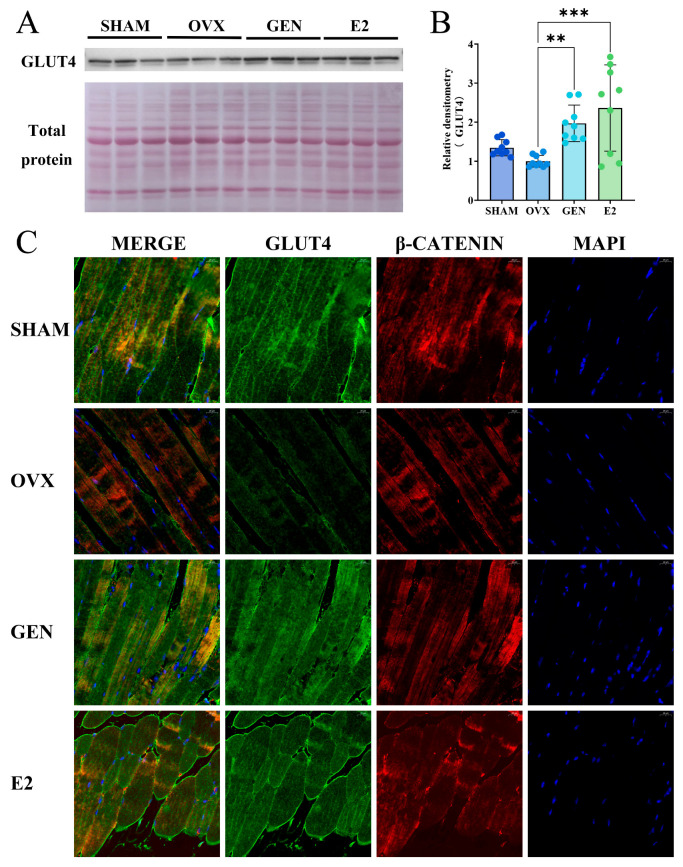
Protein expression and translocation of GLUT4 in gastrocnemius muscle. (**A**,**B**) GLUT4 protein expression (*n* = 9); (**C**) co-localization of GLUT4 with plasma membrane. GLUT4, green; plasma membrane (β-CATENIN), red; nucleus (MAPI), blue. Fluorescence micrograph acquired at 400× total magnification. ** *p* < 0.01, *** *p* < 0.001.

To verify whether the GEN-induced high GLUT4 mRNA expression (137.1%) extended to the protein level, we examined the protein expression level of GLUT4 in gastrocnemius muscle. The results showed that both GEN and E2 treatments increased GLUT4 protein expression in the gastrocnemius muscle of OVX mice, but there was no significant change in GLUT4 protein expression in OVX mice compared with SHAM mice (Figure 6A,B). Meanwhile, the co-localization signal of GLUT4 with cell membrane markers was attenuated in OVX mice compared with SHAM mice, whereas GEN and E2 treatments restored the membrane localization level of GLUT4 (Figure 6C). These results indicate that OVX did not significantly alter the total protein expression of GLUT4 but inhibited its translocation to the plasma membrane, whereas GEN and E2 not only increased the expression of GLUT4 but also promoted the translocation of GLUT4 to the plasma membrane.

### 3.6. GEN Increased GPER Expression in Gastrocnemius Muscle

**Figure 7:**
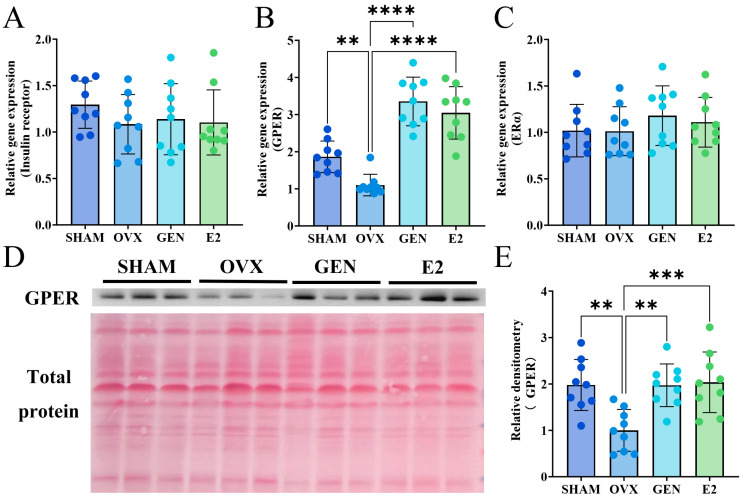
Estrogen receptors in gastrocnemius muscle. (**A**) Insulin receptors mRNA expression (*n* = 9); (**B**) GPER mRNA expression (*n* = 9); (**C**) ERα mRNA expression (*n* = 9); (**D**,**E**) GPER protein expression (*n* = 9). ** *p* < 0.01, *** *p* < 0.001, **** *p* < 0.0001.

Binding of insulin to the insulin receptor is the major pathway regulating GLUT4, but GEN did not significantly affect insulin levels (Figure 4J) or the expression of insulin receptors in gastrocnemius muscle (Figure 7A). This suggests that the effect of GEN on GLUT4 may not be through regulation of insulin secretion. GEN does not bind directly to insulin receptor but can regulate the downstream pathway of the insulin cascade response through activation of the estrogen receptor. We found that GPER expression was reduced by 83.5% in the gastrocnemius muscle of OVX mice compared with SHAM mice. GPER expression was increased by 236.0% in GEN mice and 190.4% in E2 mice compared with OVX mice (Figure 7B). Neither GEN nor E2 affected ERα expression in gastrocnemius muscle (Figure 7C). The protein expression of GPER in gastrocnemius muscle was consistent with mRNA expression (Figure 7D,E). ERβ mRNA detection was unreliable (Cp > 34) and thus omitted from statistical reporting [28].

### 3.7. GEN Increased PI3K/AKT Phosphorylation Level in Gastrocnemius Muscle

**Figure 8:**
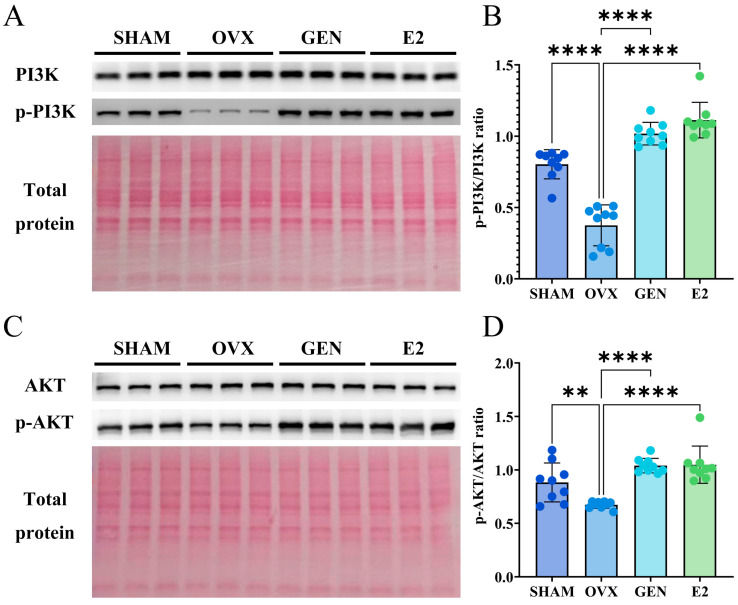
PI3K/AKT signaling pathway in gastrocnemius muscle. (**A**,**B**) PI3K and p-PI3K protein expression (*n* = 9); (**C**,**D**) AKT and p-AKT protein expression (*n* = 9). ** *p* < 0.01, **** *p* < 0.0001.

Activation of the PI3K/AKT pathway is a key signaling event that triggers GLUT4 membrane translocation and promotes cellular glucose uptake [29]. We found that compared to the SHAM group, the phosphorylation level of PI3K was reduced by 61.36% in the OVX group. In contrast, compared to the OVX group, the PI3K phosphorylation level was increased by 64.25% in the GEN group and by 73.74% in the E2 group (Figure 8A,B). Similarly, compared to the SHAM group, the phosphorylation level of AKT was decreased by 20.92% in the OVX group. Compared to the OVX group, the AKT phosphorylation level was increased by 36.69% in the GEN group and by 37.57% in the E2 group (Figure 8C,D).

### 3.8. GEN Increased Liver Glycogen but Did Not Affect Muscle Glycogen

**Figure 9:**
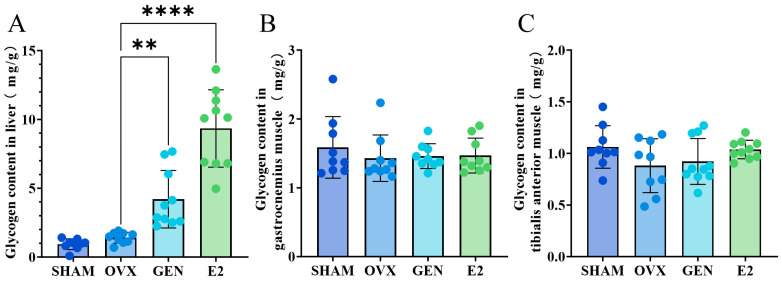
(**A**) Glycogen content of liver; (**B**) glycogen content of gastrocnemius muscle; (**C**) glycogen content of tibialis anterior muscle. ** *p* < 0.01, **** *p* < 0.0001. SHAM/OVX: *n* = 9; GEN/E2: *n* = 10.

To explore the further metabolic fate of glucose after its uptake by tissues, we examined glycogen content in liver and skeletal muscle. OVX did not affect hepatic glycogen in HFD-fed mice. E2 increased hepatic glycogen content by 2-fold in OVX mice, and GEN also increased hepatic glycogen content by 5.67-fold in OVX mice (Figure 9A). There was no significant difference in muscle glycogen levels (tibialis anterior and gastrocnemius) between the different groups (Figure 9B,C).

## 4. Discussion

Under sustained HFD conditions, GEN improved glucose metabolic homeostasis in OVX mice. This was evidenced by reductions in FBG, 2h-PG, and the homeostasis model assessment of HOMA-IR index. Concurrently, GEN upregulated the expression of GLUT4 in the gastrocnemius muscle and GLUT2 in the liver. Furthermore, GEN promoted phosphorylation of the PI3K/AKT pathway in the gastrocnemius muscle. Notably, although GEN enhanced the expression of GPER in the gastrocnemius muscle, it did not alter serum estrogen levels or uterine morphological features (uterine weights).

The present study showed that 10 weeks of GEN intervention promoted GLUT4 expression and translocation in the gastrocnemius muscle of ovariectomized mice fed a high-fat diet. However, previous studies generally suggested that GEN, as a tyrosine kinase inhibitor, downregulates GLUT4 expression and membrane translocation by inhibiting phosphorylation-dependent pathways [30,31]. The analysis of the reasons for this discrepancy involves four main aspects. Firstly, because GEN is a phytoestrogen, its physicochemical properties differ under varying estrogen levels. Previous studies on GEN inhibiting GLUT4 expression and translocation were limited to single in vitro culture systems of skeletal muscle or adipocytes. While this model offers cell-specific advantages, it cannot replicate the complex neuroendocrine regulatory network of the whole organism [32,33]. This study employed an ovariectomy model, whose metabolic disorder characteristics align more closely with the hormonal imbalance in postmenopausal women. This likely makes GEN favor activating estrogen receptor (ER)-dependent pathways by mimicking estradiol’s (E2) estrogen-like effects in skeletal muscle tissue, thereby increasing GLUT4 expression. Conversely, in in vitro single-cell models, due to the saturable shift in estrogen receptors, GEN might negatively regulate GLUT4 via kinase inhibitory pathways. The finding that GEN treatment significantly increased GPER expression in the gastrocnemius muscle of ovariectomized mice in this study supports this hypothesis. Coincidentally, a previous animal study also reported similar results, showing that GEN increased GLUT4 mRNA expression in the gastrocnemius muscle of ovariectomized Zucker diabetic fatty rats [34]. Secondly, the chronic inflammatory state and insulin resistance induced by the high-fat diet in ovariectomized mice likely increased the demand for GLUT4 in glucose metabolism. This could amplify GEN’s regulatory effect on GLUT4, leading to significant changes in its expression. Thirdly, GEN’s regulation of GLUT4 exhibits a concentration-dependent biphasic effect, making dose selection critical. In vitro studies indicate that low concentrations of GEN (0.1–1 μM) significantly enhance glucose transport and inhibit fatty acid oxidation in C2C12 myotubes, whereas high concentrations (e.g., 50 μM) produce the opposite inhibitory effect [35]. The GEN dose used in this study was 50 mg/kg/day. Pharmacokinetic data show that this dose results in a peak plasma concentration of 1.57 μM in mice after oral administration, which falls within the physiologically achievable low-concentration range [23]. It is therefore inferred that at this dose, GEN is more likely to exert a promoting effect by activating pathways such as ERs, consistent with the observed increase in GLUT4 expression and translocation. Notably, population studies indicate that plasma GEN concentrations can reach 3–4 μM after oral intake of soy milk, while concentrations after consuming soy beverages and soy extract capsules are approximately 0.429 μM and 0.966 μM, respectively [36]. This suggests that postmenopausal women can achieve the therapeutic threshold for improving glucose metabolism abnormalities through daily dietary intake, such as drinking soy milk, and that direct soy milk intake may be more effective than soy extract capsules. Finally, GEN’s effect shows significant muscle-type specificity. Its promotion of GLUT4 expression and translocation was significant in the gastrocnemius muscle, rich in slow-twitch fibers, but not obvious in the tibialis anterior muscle, rich in fast-twitch fibers. This specificity stems not only from differences in muscle fiber type composition (the tibialis anterior is predominantly composed of glycolytic fast-twitch type IIb fibers, while the gastrocnemius contains oxidative slow-twitch type I fibers and some fast-oxidative type IIa fibers) [37] but also is closely related to the inherent glucose metabolism regulatory characteristics of different fiber types. Specifically, slow-twitch fibers (type I) typically have higher basal GLUT4 expression levels, stronger insulin sensitivity, and greater insulin-stimulated glucose uptake capacity. They are rich in mitochondria, rely on oxidative phosphorylation for energy, and are crucial for maintaining blood glucose homeostasis [38]. In contrast, fast-twitch fibers (especially type IIb) rely more on glycolysis for energy and have relatively lower basal GLUT4 levels and insulin-stimulated glucose uptake capacity [39]. Furthermore, estrogen receptor (ER) expression in slow-twitch fibers may be higher than in fast-twitch fibers, providing a more favorable molecular basis for GEN to exert its effects via ER pathways in slow-twitch fibers. Interestingly, the results of a randomized controlled study indirectly support the differential responsiveness of different fiber types to specific stimuli. That study found that whey protein concentrate had the greatest effect on increasing the cross-sectional area of type II muscle fibers (Cohen’s d = 1.30), while soy protein concentrate rich in isoflavones (such as GEN) had the greatest effect on increasing the cross-sectional area of type I muscle fibers (Cohen’s d = 0.84) [40]. In this study, the gastrocnemius muscle (rich in slow-twitch fibers), under the insulin-resistant state induced by a high-fat diet and ovariectomy, likely has a more urgent demand for improved glucose metabolism. It may therefore be more sensitive to GEN’s effect of upregulating GLUT4 expression and translocation via ER pathways. Conversely, the metabolic characteristics of the tibialis anterior muscle (rich in fast-twitch fibers) and its differing responsiveness to GEN’s regulatory pathways collectively explain the lack of significant change in its GLUT4 expression.

This study demonstrates that genistein (GEN) independently enhances GLUT4 expression and membrane translocation in the gastrocnemius muscle without altering serum insulin levels or insulin receptor mRNA expression, indicating its regulation of glucose metabolism through a non-insulin-dependent mechanism. The research focuses on the estrogen receptor mechanism and reveals that GEN specifically upregulates the expression of the G protein-coupled estrogen receptor (GPER) without affecting the classical nuclear receptors ERα or ERβ. These two types of receptors exhibit distinct functional differences: ERα/ERβ primarily regulate reproductive function [41], while GPER knockout mice show no significant reproductive defects [42]. This selectivity is further supported by experimental evidence from this study, as GEN intervention did not increase uterine weight in ovariectomized mice. Notably, although no differences in GLUT4 mRNA or total protein levels were observed between the SHAM and OVX groups, immunofluorescence revealed significantly higher GLUT4 membrane localization in the SHAM group, along with higher GPER expression and PI3K/AKT phosphorylation levels compared to the OVX group. This suggests that under physiological conditions, circulating estrogen primarily regulates GLUT4 membrane translocation through the GPER/PI3K/AKT pathway. Under estrogen-deficient conditions, both GEN and E2 interventions activate this pathway (significantly increasing PI3K/AKT phosphorylation in the gastrocnemius muscle, *p* < 0.05) and synergistically promote GLUT4 expression. Numerous studies have confirmed that PI3K/AKT pathway activation is a key step in inducing GLUT4 membrane translocation [29]. Research by Pollyana Peixoto et al. further demonstrates that GPER can also mediate PI3K/AKT pathway activation [43]. This study found significantly elevated phosphorylation levels of PI3K and AKT in gastrocnemius muscle tissue (*p* < 0.05), suggesting that GEN may promote GLUT4 expression and membrane translocation by activating the GPER/PI3K/AKT pathway. Given that estrogen-related adverse effects, such as tumor risk, are primarily associated with ERα activation [44], GEN’s selective targeting of GPER may offer a higher safety profile. Although oral hormone replacement therapy (HRT) has been reported to potentially increase the risk of venous thromboembolism (VTE), and HRT may enhance vascular plaque vulnerability and thrombosis in advanced atherosclerosis, GEN has not been reported to increase such risks. On the contrary, many studies indicate its beneficial effects on cardiovascular health. For example, research by Jinxia Wu et al. [45] showed that GEN alleviates doxorubicin-induced cardiomyocyte autophagy and apoptosis in rat models via the ERK/STAT3/c-Myc signaling pathway. An analysis by Atefeh Amerizadeh et al. [46] demonstrated that genistein intake significantly reduces total cholesterol, low-density lipoprotein, and other cardiovascular disease risk factors in postmenopausal women and directly lowers systolic blood pressure in this population. In summary, GEN may serve as a targeted estrogen replacement strategy by activating the GPER/PI3K/AKT pathway to enhance GLUT4 expression and membrane translocation, thereby improving postmenopausal glucose metabolic disorders while avoiding typical estrogen-related adverse effects.

Collectively, these results position GEN as a targeted estrogen alternative for im-proving glucose metabolism disorders in postmenopausal women. It offers metabolic benefits while avoiding typical estrogenic liabilities. In addition, this study found that GEN significantly increased glucose metabolism in the liver, including upregulation of hepatic GLUT2 expression and increased hepatic glycogen content. Two studies found similar results that GEN could modulate the PKC-MAPK pathway or cAMP/PKA-AMPK to increase GLUT2 expression in hepatocytes [47,48]. OVX alone also resulted in a non-significant increase in liver GLUT2 mRNA levels (a 23.7% increase compared to the sham surgery group). Combined with the up-regulation of GLUT4 in skeletal muscle and the enhanced membrane translocation, this suggests that GEN has a “multi-organ syner-gistic” regulatory property, i.e., systemic glucose homeostasis is improved through the dual optimization of skeletal muscle (glucose uptake) and liver (glycogen storage). This multi-targeted mode of action provides a unique advantage for GEN in the treatment of postmenopausal metabolic syndrome.

Having clarified that GEN can activate GPER to modulate GLUT4 to enhance gastrocnemius muscle glucose uptake, the present study assessed the effect of GEN on gastrocnemius muscle glycogen storage capacity by assaying glycogen. Interestingly, neither OVX, GEN, nor E2 significantly induced changes in gastrocnemius muscle glycogen content, suggesting that increased glucose uptake may be preferentially directed to other metabolic pathways (aerobic oxidative pathway or lactic acid generating pathway) resulting in greater conversion of glucose to ATP rather than storage as glycogen. In addition, previous studies have shown that high-fat diets inhibit glycogen synthase activity, leading to deficient glycogen synthesis, and while GEN enhances glucose uptake in the gastrocnemius muscle, HFD-induced inhibition of muscle glycogen synthesis persists, and estrogen levels do not have a significant effect on this inhibition.

Furthermore, adipose tissue plays a critical role in glucose metabolism and insulin resistance. Under normal physiological conditions, fat is preferentially stored as subcutaneous adipose tissue; however, when energy intake is excessive, excess lipids are more likely to accumulate in intra-abdominal visceral adipose tissue. Intra-abdominal obesity has been clearly demonstrated to be independently associated with an increased risk of insulin resistance, diabetes, hypertension, and cardiovascular disease, irrespective of the degree of overall obesity [49]. Previous studies have shown that postmenopausal women have a significantly higher prevalence of intra-abdominal fat accumulation compared to premenopausal women [50]. This study observed that although there were no statistically significant differences in body weight changes between the GEN or E2 groups and the SHAM group (*p* > 0.05), both the GEN and E2 groups showed a significant reduction in abdominal fat mass compared to the SHAM group (*p* < 0.05). Concurrently, the E2 group exhibited a significant increase in food intake (*p* < 0.05). These results suggest that both E2 and GEN can effectively improve body fat distribution in ovariectomized (OVX) mice, particularly by reducing abdominal fat accumulation, even though this improvement was not directly reflected in a significant decrease in overall body weight. The discrepancy between reduced abdominal fat and unchanged body weight may be related to the increased food intake induced by E2. The relationship between postmenopausal hormone therapy and body weight changes remains debated; however, it is now generally believed that obesity assessment should focus more on fat distribution rather than total body weight. Even in cases where body weight increases, a more uniform distribution of fat (as opposed to centralized visceral deposition) may still represent a healthier overall adiposity profile [20].

This study has several limitations. First, although we observed increased GLUT4 expression and membrane translocation in the gastrocnemius muscle, along with upregulated GPER expression and elevated phosphorylation levels of PI3K and AKT, we did not employ specific inhibitors to block critical nodes of this pathway to further validate its necessity. Nevertheless, previous research supports the scientific plausibility that GEN enhances GLUT4 translocation and facilitates glucose uptake in the gastrocnemius muscle via the GPER/PI3K/AKT pathway. We intend to further explore this mechanism in future studies by examining upstream and downstream genes and proteins to consolidate the signaling cascade. Second, based on statistical considerations and in compliance with animal ethics principles aimed at reducing unnecessary animal use, this study did not include a SHAM surgery group supplemented with GEN intervention. Existing studies indicate that GEN can still improve glucose metabolism in non-estrogen deficient models. For example, Schuyler Rockwood et al. found that while GEN did not alter serum insulin levels in leptin deficient ob/ob mice, it significantly reduced their blood glucose levels. Similarly, Nana Zhang et al. reported that GEN administered at 60 mg/kg markedly improved insulin resistance in mice fed a high fat diet. These results suggest that GEN retains the potential to modulate glucose metabolism even in the absence of estrogen deficiency. However, there is currently no direct evidence demonstrating whether GEN can promote GLUT4 expression in skeletal muscle, specifically in the gastrocnemius, of female mice with normal estrogen levels. Therefore, we plan to include a SHAM + GEN group in future experiments to clarify the effects of GEN on GLUT4 expression and function in skeletal muscle across varying endogenous estrogen levels.

## 5. Conclusions

In summary, this study demonstrates that under estrogen-deficient conditions, GEN concurrently upregulates both GPER and GLUT4 expression in the gastrocnemius muscle while facilitating GLUT4 translocation to the plasma membrane through enhanced PI3K/AKT phosphorylation. Simultaneously, GEN elevates hepatic GLUT2 expression and stimulates glycogen synthesis. Our investigation focused primarily on two key aspects. First, by assessing GLUT expression across glucose-metabolizing tissues, we identified the gastrocnemius muscle as GEN’s primary target for ameliorating glucose metabolic disorders in ovariectomized mice, showing significantly greater regulatory efficacy than the tibialis anterior muscle and liver tissue. Second, under high-fat diet conditions, GEN activates the GPER/PI3K/AKT pathway to promote GLUT4 expression and membrane translocation specifically in the gastrocnemius muscle of ovariectomized mice.

## Figures and Tables

**Table 1:** Experimental group design and interventions.

Group	Feeding Method	Surgical Treatment	Intervention Method	Intervention Dose
SHAM	HFD	sham surgery	oral gavage	corn oil, 0.2 mL/day
OVX	HFD	bilateral oophorectomy	oral gavage	corn oil, 0.2 mL/day
GEN	HFD	bilateral oophorectomy	oral gavage	GEN, 50 mg/kg/day
E2	HFD	bilateral oophorectomy	subcutaneous injections	E2, 50 μg/kg twice weekly

Genistein (GEN) and 17β-estradiol (E2) are both soluble in corn oil.

**Table 2:** Primer sequences for quantitative real-time PCR.

Gene	Sequences
β-actin F	GAGCGCAAGTACTCTGTGTG
β-actin R	AACGCAGCTCAGTAACAGTC
GLUT2 F	ACCGGGATGATTGGCATGTT
GLUT2 R	GGACCTGGCCCAATCTCAAA
GLUT4 F	GCTCTGACGTAAGGATGGGGAAC
GLUT4 R	CAATCACCTTCTGTGGGGCAT
ERα F	CCTCCCGCCTTCTACAGGT
ERα R	CACACGGCACAGTAGCGAG
ERβ F	TTTGTGGAGCTCAGCCTGTT
ERβ R	CTCATCCCTGTCCAGAACGA
GPER F	ACGCCACGGCACAGATCA
GPER R	TTCTGTCCTTGGTCCAGATCG
Insulin receptor F	AAAAACCTCTTCAGGCAATGGTG
Insulin receptor R	GTCACATTCCCCACCTCTTCA

**Table 3:** Body weight and organ mass.

Parameter	SHAM (*n* = 9)	OVX (*n* = 9)	GEN (*n* = 10)	E2 (*n* = 10)	*p* Value
Body weight					
Initial weight (g)	19.81 ± 0.585	19.49 ± 0.480	19.62 ± 0.480	19.50 ± 0.725	0.7658
Final weight (g)	22.35 ± 1.393 *	26.81 ± 3.239	25.76 ± 1.768	27.97 ± 0.740	<0.0001
Abdominal fat (g)	0.587 ± 0.173 *	1.526 ± 0.690	1.049 ± 0.435 *	0.605 ± 0.161 *	<0.0001
Gastrocnemius muscle (g)	0.238 ± 0.011 *	0.260 ± 0.015	0.258± 0.017	0.265 ± 0.008	0.0008
Tibialis anterior muscle (g)	0.079 ± 0.009	0.085 ± 0.013	0.092 ± 0.013	0.112 ± 0.008 *	<0.0001
Pancreas (g)	0.122 ± 0.024	0.126 ± 0.026	0.139 ± 0.021	0.167 ± 0.038 *	0.0060
Liver (g)	0.910± 0.208	0.839 ± 0.087	0.893 ± 0.068	1.063 ± 0.065 *	0.0017

* *p* < 0.05, vs. OVX.

## Data Availability

The original contributions presented in this study are included in the article. Further inquiries can be directed to the corresponding author.

## Abbreviations

The following abbreviations are used in this manuscript:

2h-PG	2 h postprandial glucose
ANOVA	analysis of variance
AUC	area under the curve
E2	17β-estradiol
FBG	fasting blood glucose
GAPDH	glyceraldehyde-3-phosphate dehydrogenase
GEN	genistein
GLUT4	glucose transporter 4
GPER	G protein-coupled estrogen receptor
HFD	high-fat diet
HOMA	homeostasis model assessment
HRT	hormone replacement therapy
IRS1	insulin receptor substrate
ITT	insulin tolerance test
OGTT	oral glucose tolerance test
OVX	ovariectomized
qPCR	quantification real-time polymerase chain reaction
P-IRS1	phosphorylated insulin receptor substrate
T2D	type 2 diabetes
VTE	venous thromboembolism
